# Study on Bifurcation and Dual Solutions in Natural Convection in a Horizontal Annulus with Rotating Inner Cylinder Using Thermal Immersed Boundary-Lattice Boltzmann Method

**DOI:** 10.3390/e20100733

**Published:** 2018-09-25

**Authors:** Yikun Wei, Zhengdao Wang, Yuehong Qian, Wenjing Guo

**Affiliations:** 1 Faculty of Mechanical Engineering & Automation, Zhejiang Sci-Tech University, Hangzhou 310018, China; 2 State-Province Joint Engineering Lab of Fluid Transmission System Technology, Hangzhou 310018, China; 3 Shanghai Institute of Applied Mathematics and Mechanics, Shanghai University, Shanghai 200072, China; 4 School of Mathematical Sciences, Soochow University, Suzhou 215006, China; 5 Basic Courses Department, Shandong University of Science and Technology, Taian 271019, China

**Keywords:** natural convection, bifurcation, horizontal annulus, thermal IB-LBM, rotation

## Abstract

A numerical investigation has been carried out to understand the mechanism of the rotation effect on bifurcation and dual solutions in natural convection within a horizontal annulus. A thermal immersed boundary-lattice Boltzmann method was used to resolve the annular flow domain covered by a Cartesian mesh. The Rayleigh number based on the gap width is fixed at 10^4^. The rotation effect on the natural convection is analyzed by streamlines, isotherms, phase portrait and bifurcation diagram. Our results manifest the existence of three convection patterns in a horizontal annulus with rotating inner cylinder which affect the heat transfer in different ways, and the linear speed ($U_{i}^{*}$) determines the proportion of each convection. Comparison of average *Nusselt* number versus linear speed for the inner cylinder indicates the existence of the three different mechanisms which drive the convection in a rotation system. The convection pattern caused by rotation reduces the heat transfer efficiency. Our results in phase portraits also reveal the differences among different convection patterns.

## 1. Introduction

Natural convection in a horizontal annulus is a useful subject due to its importance in theoretical study and in many engineering applications, such as thermal energy storage systems, cooling systems in electronic components, brake assembly and bearing fittings. Considering its simple geometry, this system has become a classical model and has been well studied by many researchers. Early in 1966, Grigull and Hauf [1] investigated natural convection in a horizontal annulus experimentally. In 1969, Powe et al. [2] designed an experiment and delineated the convection with different types of flow depending on the Grashof number and radius ratio. Kuehn and Goldstein [3] studied the natural convection in concentric cylinders numerically and experimentally. Nguyen et al. [4] presented the flow patterns and heat transfer rates in terms of the radius ratio, the Rayleigh number and inversion parameter. Dyko et al. [5] presented a three-dimensional numerical and experimental study for flow between two horizontal coaxial cylinders at Rayleigh numbers approaching and exceeding the critical values. The instability and bifurcation phenomena in the natural convection in a horizontal annulus have also attracted the interest of many researchers. Yoo et al. [6,7,8] studied the effect of the Prandtl number on the bifurcation phenomenon of natural convection in an annulus with a narrow or wide gap. Results show that natural convection with high Prandtl number in a narrow annulus has bifurcation solutions. Petrone et al. [9,10] analyzed the bifurcation phenomena theoretically through a bifurcation diagram by numerical simulation. Luo et al. [11] reported the eccentricity effect on the dual solutions. Hu et al. [12] demonstrated three solutions in the wide gap annulus with a constant heat flux at wall. Zhang et al. [13] investigated the natural convection in a circular enclosure partitioned by a thin flat plate. Natural convection in other types of enclosures has also been studied by many other researchers [14,15].

It has been confirmed that the rotation effect plays a significant role in natural convection in an annulus in applications such as the heat transfer in bearing system, i.e., in the work of Lopez et al. [16] which studied the rotation effect on convection in a vertical annulus. For a square enclosure within which there is a rotating circular cylinder, Zhang et al. [17] found that the heat transfer can be conduction-dominated or mixed convection-dominated depending on the rotating speed. In this paper, we focus on the rotation effect on the natural convection in a horizontal annulus. The mechanism of the rotation effect is quantified by linear speed of rotational inner cylinder, and is presented and analyzed by the streamlines and isotherms at different dimensionless linear speeds. We also use the bifurcation diagram of rotation system to discuss the bifurcation solutions of natural convection in a horizontal annulus with stationary inner and outer cylinders. The present study of the rotation effect on natural convection provides a thorough insight in understanding the heat transfer in the bearing system problem.

All simulations in this study are carried out using a thermal immersed boundary-lattice Boltzmann method (IB-LBM). The lattice Boltzmann method (LBM) has been widely adopted in computational fluid dynamics for its convenience in treating curved boundaries and performing parallel computation [12,14,15,16,18,19,20,21,22,23,24,25,26,27,28,29,30]. It exhibits great convenience in heat and mass transfer [6,7,8,12]. The IB-LBM has achieved great success in dealing with moving boundaries with complex geometry since 2004 since Feng et al. [21] first introduced the IBM into LBM. Since then, a lot of efforts have been made to improve the accuracy of this method [22,24,25,26]. Feng et al. [22] used the idea of direct force to calculate the boundary force. Niu et al. [24] considered the moment exchange on the boundary. Wu et al. [25,26] proposed an implicit velocity correction-based model. For thermal fluids, researchers made efforts to deal with the thermal boundary conditions [12,28,29,30]. Hu et al. [12] developed a thermal IB-LBM to simulate both the temperature Dirichlet boundary condition and the Neumann boundary condition. Wu et al. discussed the accuracy of thermal IB-LBM [30]. Recently, the thermal lattice Boltzmann method with curving boundary treatment becomes popular in simulating natural convection in a horizontal annulus [11,12,31,32].

The remainder of this paper is organized as follows. Section 2 describes the scope of simulations and briefly introduces the numerical method used in this paper. In Section 3, numerical results are compared with experiments. In Section 4, numerical results and the effect of rotation on the natural convection are presented. We summarize our findings and conclude in Section 5.

## 2. Numerical Model and Method

In this section, the configuration of natural convection in a horizontal annulus and a treatment for thermal flow with curved boundaries are introduced. We follow the idea of the coupled lattice BGK model (CLBGK) [21] to treat thermal flow field. The IB-LBM is used to treat the boundary conditions.

### 2.1. Numerical Model

**Figure 1:**
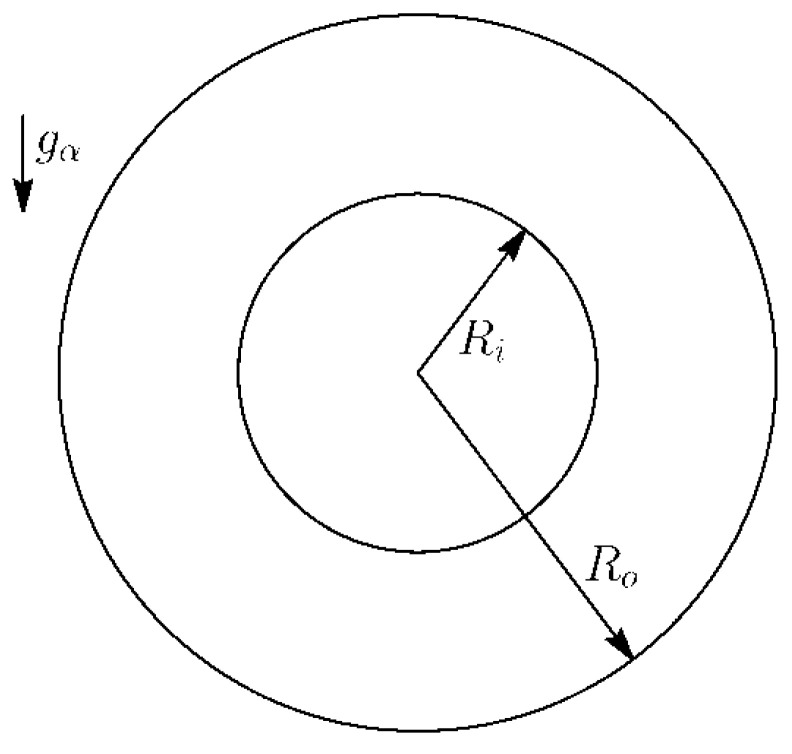
Configuration of natural convection in a horizontal annulus.

The configuration of a two-dimensional natural convection is shown in Figure 1. $R_{i}$ and $R_{o}$ denote the radius of inner and outer cylinders, respectively. The flow is driven by gravity $G_{\alpha }$. The governing equations of the convection are [23]:

(1) $$\begin{array}{cc} \partial_{t}\rho +\partial_{\alpha }\left(\rho u_{\alpha }\right)=0 & \left(a\right)\\ \partial_{t}\left(\rho u_{\alpha }\right)+\partial_{\beta }\left(\rho u_{\alpha }u_{\beta }\right)=G_{\alpha }+F_{\alpha }-\partial_{\alpha }p+\partial_{\beta }\left[\mu \left(\partial_{\alpha }u_{\beta }+\partial_{\beta }u_{\alpha }\right)\right] & \left(b\right)\\ \partial_{t}T+u_{\alpha }\partial_{\alpha }T=\kappa \partial_{\alpha }^{2}T+\Phi & \left(c\right) \end{array}$$

where ρ is the fluid density, $u_{\alpha }$ is velocity, $G_{\alpha }$ is the buoyancy, $F_{\alpha }$ is the body force, μ is the dynamic viscosity, T is the temperature, κ is thermal diffusivity and Φ is the heat source term. The inner and outer cylinder surfaces are maintained at different uniform temperatures $T_{h}$ and $T_{c}$($T_{h}>T_{c}$), respectively. With the Boussinesq approximation [21], the fluid density is assumed to be a linear function of the temperature and the buoyancy can be written as: $G_{\alpha }=-g_{\alpha }\beta \left(T-T_{0}\right)$ and β is the coefficient of thermal expansion and $T_{0}$ is the average temperature of fluid.

Some dimensionless variables in natural convection are introduced as follows. The Mach number (Ma):

(2) $$Ma=\frac{\sqrt{|g_{\alpha }|\beta \Delta TL}}{c_{s}},$$

where $\Delta T=T_{h}-T_{c}$ is temperature difference between hot wall and cold wall, L is the characteristic length across the temperature field which is $R_{o}-R_{i}$ for the convection in a horizontal annulus. The Mach number used in present work is fixed at 0.01 to ensure that the flow is incompressible.

The Rayleigh number (Ra):

(3) $$Ra=\frac{|g_{\alpha }|\beta \Delta TL^{3}}{\nu \kappa }=\frac{{\left(MaLc_{s}\right)}^{2}Pr}{\nu^{2}},$$

where Pr is the Prandtl number defined as Pr=ν/κ.

The *Nusselt* number (Nu):

(4) $$Nu=\frac{\left|q_{\alpha }\right|}{Q_{c}}=\frac{\left|u_{\alpha }T-\kappa \partial_{\alpha }T\right|}{Q_{c}},$$

where $\left|q_{\alpha }\right|$ is the quantity of local heat flux and $Q_{c}$ is the average heat flux resulting from pure conduction.

### 2.2. Thermal IB-LBM

To solve the governing equations, the CLBGK model [23] is used in this paper. The general philosophy is to solve the velocity field and the temperature field using two independent lattice BGK equations, respectively, and then to combine them into one coupled model for the Boussinesq incompressible flow.

The lattice BGK equation with external force term reads:

(5) $$f_{i}\left(x_{\alpha }+c_{i\alpha }\delta_{t},t+\delta_{t}\right)-f_{i}\left(x_{\alpha },t\right)=-\omega_{f}\left[f_{i}\left(x_{\alpha },t\right)-f_{i}^{\left(eq\right)}\left(x_{\alpha },t\right)\right]+F_{i}\delta_{t},$$

where $f_{i}$ is the density distribution function, $f_{i}^{\left(eq\right)}$ is the equilibrium distribution function, $c_{i\alpha }$ is the ith discretized velocity vector and $\omega_{f}$ is the relaxation parameter. For the D2Q9 model [18], the equilibrium function is:

(6) $$f_{i}^{\left(eq\right)}\left(x_{\alpha },t\right)=w_{i}\rho \left[1+\frac{c_{i\alpha }v_{\alpha }}{c_{s}^{2}}+\frac{v_{\alpha }v_{\beta }}{2c_{s}^{2}}\left(\frac{c_{i\alpha }c_{i\beta }}{c_{s}^{2}}-\delta_{\alpha \beta }\right)\right],$$

where sound speed $c_{s}$ and the weight coefficient $w_{i}$ are:

(7) $$c_{s}=\frac{1}{\sqrt{3}}\frac{\delta_{x}}{\delta_{t}},\;\;\;w_{i}=\left\{ \begin{array}{ll} 4/9 & i=0\\ 1/9 & i=1\sim 4\\ 1/36 & i=5\sim 8 \end{array}\right..$$

Using Chapman-Enskog expansion till the second order of approximation and considering the conserved quantity $\rho =\sum f_{i}$ and $\rho u_{\alpha }=\sum f_{i}c_{i\alpha }+F_{\alpha }\delta_{t}/2$, the relation between the relaxation parameter $\omega_{f}$ and kinematic viscosity ν can be obtained as:

(8) $$\nu =c_{s}^{2}\left(\frac{1}{\omega_{f}}-\frac{1}{2}\right)\delta_{t}.$$

The force scheme proposed by Buick [27] is applied in this work:

(9) $$F_{i}=w_{i}\left(1-\frac{\omega_{f}}{2}\right)\frac{G_{\alpha }c_{i\alpha }}{c_{s}^{2}},$$

where $G_{\alpha }$ is the external force.

Coupling temperature field to flow field, the well-known Boussinesq approximation is often used in the study of natural convection. With the Boussinesq approximation, the gravity can be rewritten as:

(10) $$G_{y}=-\rho |g_{\alpha }|\left[1-\beta \left(T-T_{0}\right)\right].$$

To simplify this formula, $T_{0}$ is chosen as −1/β and the gravity is:

(11) $$G_{y}=\rho |g_{\alpha }|\beta T.$$

For the flow field, non-slip boundary condition is considered in all simulations. The direct force IB-LBM proposed by Feng [22] is applied to realize the dynamical boundary condition. The discretion of the derivative of time in above equation is related to the desired boundary condition (Equation (12)). This method is also termed as direct forcing model by Wu [25] penalty method by Feng et al. [21] and direct forcing method by Feng et al. 2005 [22]. They proposed a direct force term to realize the no-slip boundary condition as formed below.

(12) $$F_{\alpha }=\rho \left(\partial_{t}u_{\alpha }+u_{\beta }\partial_{\beta }u_{\alpha }\right)-\mu \partial_{\beta }^{2}u_{\alpha }+\partial_{\alpha }p,$$

where $\partial_{t}u_{\alpha }=\frac{u_{\alpha }^{D}-u_{\alpha }}{\delta_{t}}$ and $u_{\alpha }^{D}$ is the desired value of velocity on the boundary.

Based on their method, the force term used in a thermal IB-LBM has the following formula:

(13) $$F_{\alpha }=\rho \left(\partial_{t}u_{\alpha }+u_{\alpha }\partial_{\beta }u_{\beta }\right)-\partial_{\beta }^{2}u_{\alpha }+\partial_{\alpha }p-\rho |g_{\alpha }|\beta Te_{y\alpha },$$

where $e_{y\alpha }$ is the unit vector in the *y* direction.

To simulate the thermal field, we follow the idea of CLBGK proposed by Guo [23] and use a 9-discrete-velocity model with the distribution function of temperature $g_{i}$.

(14) $$g_{i}\left(x_{\alpha }+c_{i\alpha }\delta_{t},t+\delta_{t}\right)-g_{i}\left(x_{\alpha },t\right)=-\omega_{g}\left[g_{i}\left(x_{\alpha },t\right)-g_{i}^{\left(eq\right)}\left(x_{\alpha },t\right)\right]+\Phi_{i}\delta_{t}\;$$

where $\Phi_{i}$ is discretized heat source term which is related to heat source Φ. The thermal equilibrium function is:

(15) $$g_{i}^{\left(eq\right)}\left(x_{\alpha },t\right)=w_{i}T\left[1+\frac{c_{i\alpha }v_{\alpha }}{c_{s}^{2}}+\frac{v_{\alpha }v_{\beta }}{2c_{s}^{2}}\left(\frac{c_{i\alpha }c_{i\beta }}{c_{s}^{2}}-\delta_{\alpha \beta }\right)\right].$$

To compare with Equation (1) (c), the Chapman-Enskog expansion is also used to decide the relation between $\omega_{g}$ and κ, which is:

(16) $$\kappa =c_{s}^{2}\left(\frac{1}{\omega_{g}}-\frac{1}{2}\right)\delta_{t}.$$

Following the idea of previous direct force model and considering $u_{\alpha }\partial_{\alpha }T=0$ at boundary, the isothermal boundary condition is realized by the following modified heat source:

(17) $$\Phi =\partial_{t}T-\kappa \partial_{\alpha }^{2}T\;$$

where $\partial_{t}T$ is calculated by $\partial_{t}T=\frac{T^{D}-T}{\delta_{t}}$ and $T^{D}$ is the desired value of temperature on the boundary.

Since LBM deals with Eulerian flow points and boundaries are represented by Lagrangian points, Delta function is used to exchange the message between Eulerian points and Lagrangian points and has the following formula:

(18) $$\begin{array}{l} D\left(x_{\alpha }-x_{0\alpha }\right)=\delta \left(x-x_{0}\right)\delta \left(y-y_{0}\right)\\ \delta \left(r-r_{0}\right)=\left\{ \begin{array}{ll} \frac{1}{4}\left[1+\cos\left(\frac{\pi |r|}{2}\right)\right] & |r|\leq 2\\ 0 & |r|>2 \end{array}.\right. \end{array}$$

The messages from both velocity field and thermal field are exchanged by Delta function mentioned in the above section.

## 3. Verification

The accuracy of the present method in natural convection in an annulus is verified. To qualify the results, the average *Nusselt* number ($\bar{Nu}$)is defined by the following equation:

(19) $$\bar{Nu}=\frac{{\bar{q}}_{n}}{Q_{c}}=\frac{\int q_{n}ds}{Q_{c}S}\;$$

where S is the area of the flow filed. For pure conduction, the exact solution of the temperature field in an annulus is:

(20) $$\frac{T-T_{c}}{\Delta T}=\frac{\ln\frac{R_{o}}{r}}{\ln R_{\beta }}\;$$

where radius ratio $R_{\beta }=\frac{R_{o}}{R_{i}}$. Considering the definition of the average *Nusselt* number, its formula in an annulus yields:

(21) $$\bar{Nu}=\frac{\int q_{n}ds}{Q_{c}S}=\gamma \frac{L\int q_{r}rdrd\theta }{\kappa \Delta T\pi S}=\gamma \frac{\int q_{r}rdrd\theta }{\kappa \Delta T\pi \left(R_{o}+R_{i}\right)}\;$$

where the modified parameter γ is:

(22) $$\gamma =\frac{\ln R_{\beta }\left(R_{\beta }+1\right)}{2\left(R_{\beta }-1\right)}.$$

**Figure 2:**
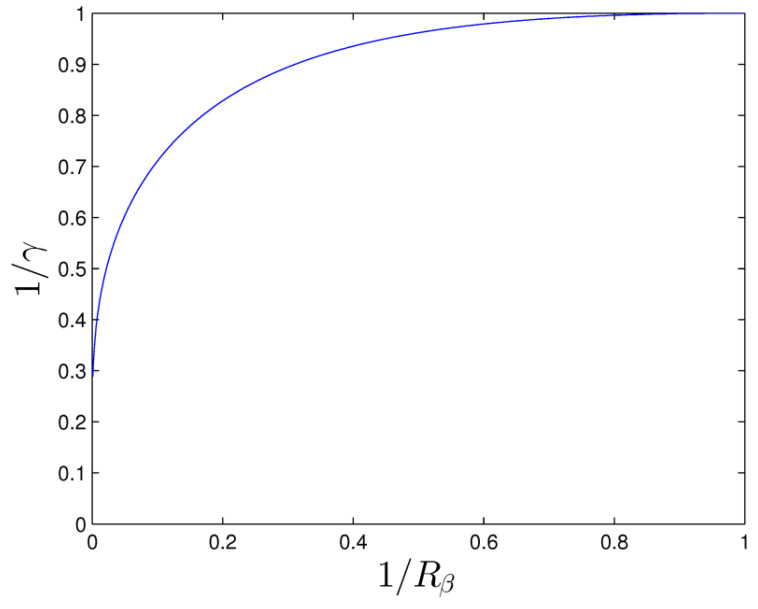
The distribution of the modified parameter γ versus the radius ratio $R_{\beta }$.

The relation between γ and $R_{\beta }$ is shown in Figure 2.

The grid independence tests of present thermal IB-LBM for natural convection in horizontal annuli are conducted. The grid sizes of 128×128, 256×256 and 512×512(l.u.) are tested for 4 different Rayleigh numbers, namely, *Ra* = 10^2^, 10^3^, 10^4^ and 2 × 10^4^ to verify the grid independence and the accuracy of the boundary treatment by comparing with the results from experiments [3]. Table 1 presents the results of average *Nusselt* number. It is shown that numerical results of the present method are in excellent agreement with experimental results. The relative errors between grid size of 256 × 256(*l.u.*) and extrapolated results are only 1.39%, 1.02%, 1.29% and 1.17% for $Ra=10^{2}$, $10^{3}$, $10^{4}$ and $5\times 10^{4}$, respectively. Results show that the grid size of 256×256(l.u.) which is used in following studies is fine enough.

## 4. Results and Discussion

In this section, the rotation effect on bifurcations of natural convection is analyzed with Pr=0.7 and $Ra=10^{4}$ in an annulus with its radius ratio $R_{\beta }=1.5$. The streamlines, isotherms and phase portrait of average *Nusselt* number will be further analyzed. In order to study the rotation effect of the inner cylinder, the linear speed of the inner cylinder is non-dimensionalized by the characteristic speed $U_{0}=\sqrt{|g_{\alpha }|\beta \Delta TL}$. As a matter of convenience, the *Nusselt* number Nu is used to represent the average *Nusselt* number in the flow field $\bar{Nu}$ in the following section.

### 4.1. Bifurcation Solutions in Stationary Concentric Cylinders

**Figure 3:**
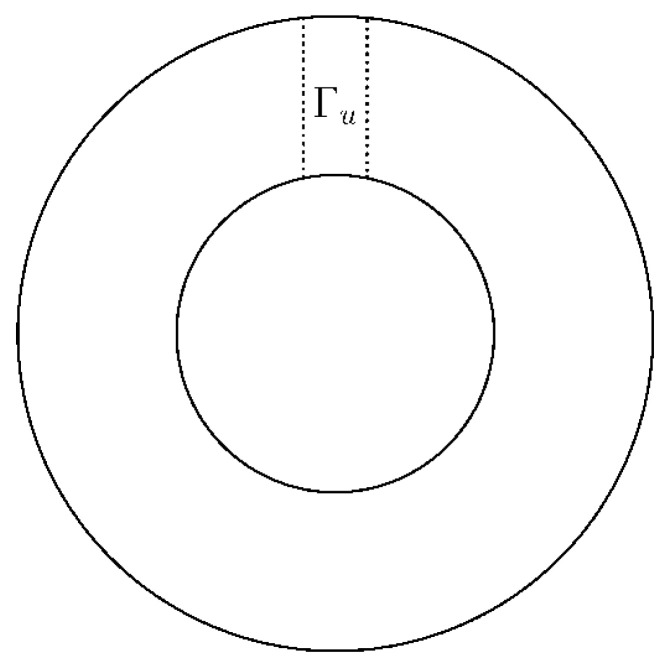
Schematic of initial condition of bifurcation solutions.

**Figure 4:**
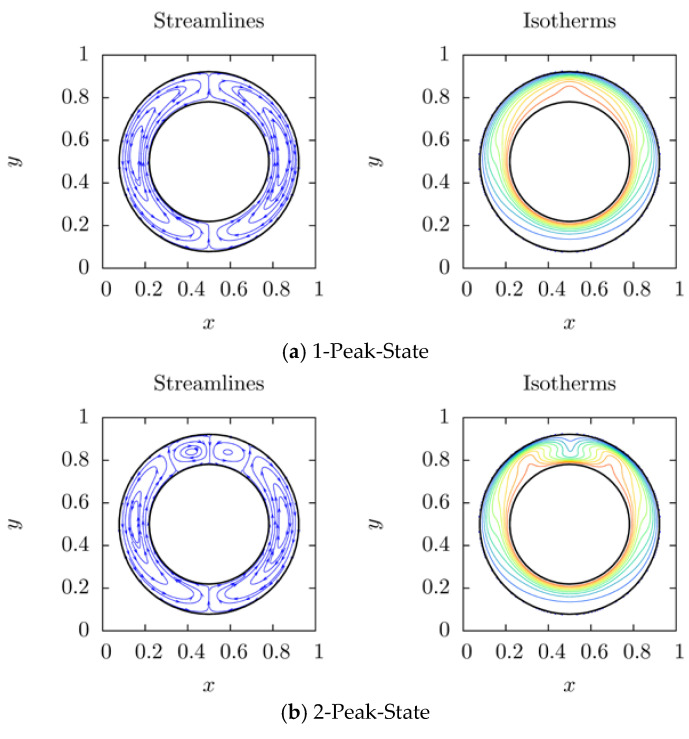

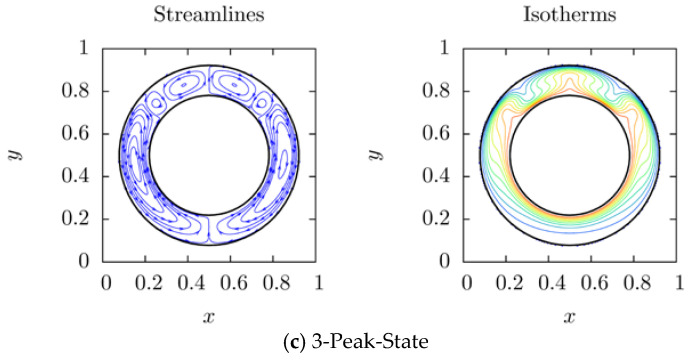
Different states at the same Ra, Pr and $R_{\beta }$. $Ra=10^{4}$, Pr=0.7, $R_{\beta }=1.5$.

It has been found by many researchers that the bifurcation phenomenon appears in the natural convection in a horizontal annulus [7,11,12,31]. Different initial conditions will lead to different structures of convection. The schematic of different initial conditions used in our paper is shown in Figure 3. With different temperature distributions at $\Gamma_{u}$, we obtained three different structure of convection. From isotherms, different numbers of hot peaks can be found in different states. In Figure 4c, for instance, one can find three hot peaks at (0.2, 0.65), (0.5, 0.8) and (0.8, 0.65), respectively. A number of hot peaks are used to distinguish these different states in this paper. In this subsection, the bifurcation phenomena are simulated, which mainly verify the accuracy of the thermal IB-LBM treatment and obtain the initial conditions for following rotating cases.

The bifurcation solutions of natural convection in a horizontal annulus with stationary inner and outer cylinders are presented. We first use zero velocity and low temperature for all flow fields as zero initial condition and obtained 1-peak state. Then 2-peak state is obtained by using the final state of 1-peak state by removing internal energy step as its initial condition. Finally, 3-peak state is obtained by using the final state of 2-peak state with an injecting internal energy step as its initial condition. The treatment of removing/injecting internal energy step in dealing with the temperature field at top region $\Gamma_{u}$ is shown in Figure 3. When implementing removing internal energy step, temperature at top region $\Gamma_{u}$ is set as the lowest temperature $T_{c}$, compulsively. The injecting internal energy step is to set the temperature at top region the highest temperature $T_{h}$, compulsively.

**Figure 5:**
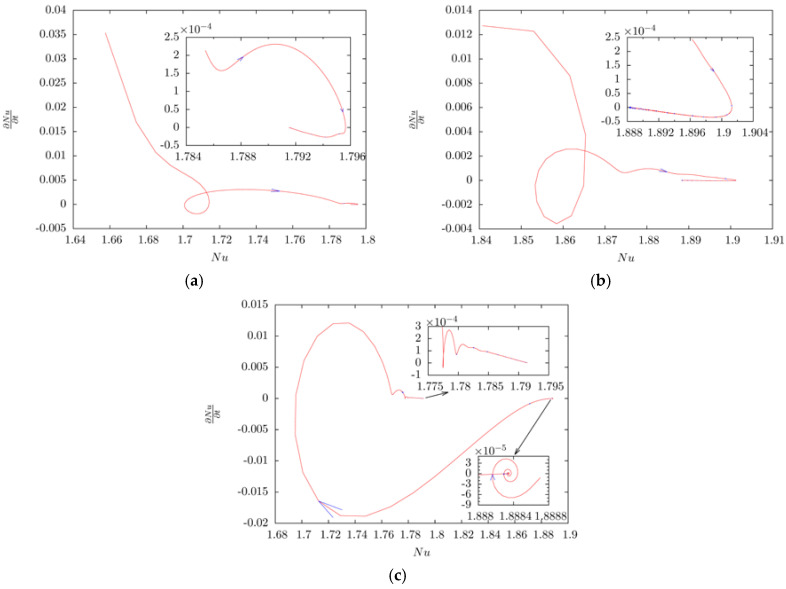
The phase portrait of Nusselt number. (**a**) Flow state turns from 1-peak-state to 2-peak-state after removing energy step; (**b**) Flow state turns from 2-peak-state to 3-peak-state after injecting energy step; (**c**) Flow state turns from 3-peak-state back to 2-peak-state after a small perturbation introduced into the system.

Figure 4 shows the results of streamlines and isotherms at $Ra=10^{4}$, Pr=0.7, $R_{\beta }=1.5$. For 1-peak-state which is shown in Figure 4a, it is observed with zero initialization. It is seen that a pair of symmetrical vortices are formed in the annulus because of the buoyancy and one hot peak in the isotherms appears at the top region. According to the formula of local *Nusselt* number (Equation (4)), it is larger at top region than the remained region. After removing internal energy step, another solution with two hot peaks of this system is observed in Figure 4b. It is seen that a new pair of vortices appear at the top region of the annulus and velocity at middle of the top region turns from upwards to downwards. Injecting internal energy to $\Gamma_{u}$ region at 2-peak state, another bifurcation consists of three hot peaks branches. 3-peak-state is presented in Figure 4c. A new pair of vortices appears at the top region. It is further observed that new vortex pair stretches and compresses the previous vortex pair and the previous vortex pair turns into two small vortices which beside the newly appeared large vortex pair. One also sees that 3-peak-state is very unstable from Figure 5c. This also suggests that with a small perturbation (inner cylinder rotate clockwise with its dimensionless linear speed exceeds 0.03), the flow state turns from 3-peak-state to 2-peak-state. It is further found that new pairs of vortices constantly appear at the top region of the annulus and the *Nusselt* number gradually increases with increasing number of hot peaks at the same Ra, Pr and $R_{\beta }$, which are 1.579, 1.791 and 1.888, respectively.

The phase portrait of *Nusselt* number is shown in Figure 5. In order to show the detail in the phase portrait, final phase trajectory of *Nusselt* number are zoomed in and presented in the inset of Figure 5. From Figure 5a,b, the phase portraits indicate that the *Nusselt* number increases with increasing hot peaks. Since the configuration is symmetric, the phase portraits in both Figure 5a,b indicate that the 2-peak-state and 3-peak-state are both stable. However, as discussed above, 3-peak-state will be unstable when the symmetry of the system breaks. Figure 5c shows the phase trajectory of the progress when a small perturbation is introduced into 3-peak-state. It is seen that any perturbation which breaks the symmetry of the system makes one of two previous small vortices compressed, the average *Nusselt* number decreases, and the compressed vortex finally disappears. Two large vortices beside the disappeared small vortex then merged into one vortex and the system turns to 2-peak-state. One also sees that the average *Nusselt* number of the newly formed 2-peak-state is smaller than that of the equilibrium system, vortices then resize under the buoyancy and the average *Nusselt* number increases till the system reaches equilibrium state.

### 4.2. Rotation Effect on Natural Convection

**Figure 6:**
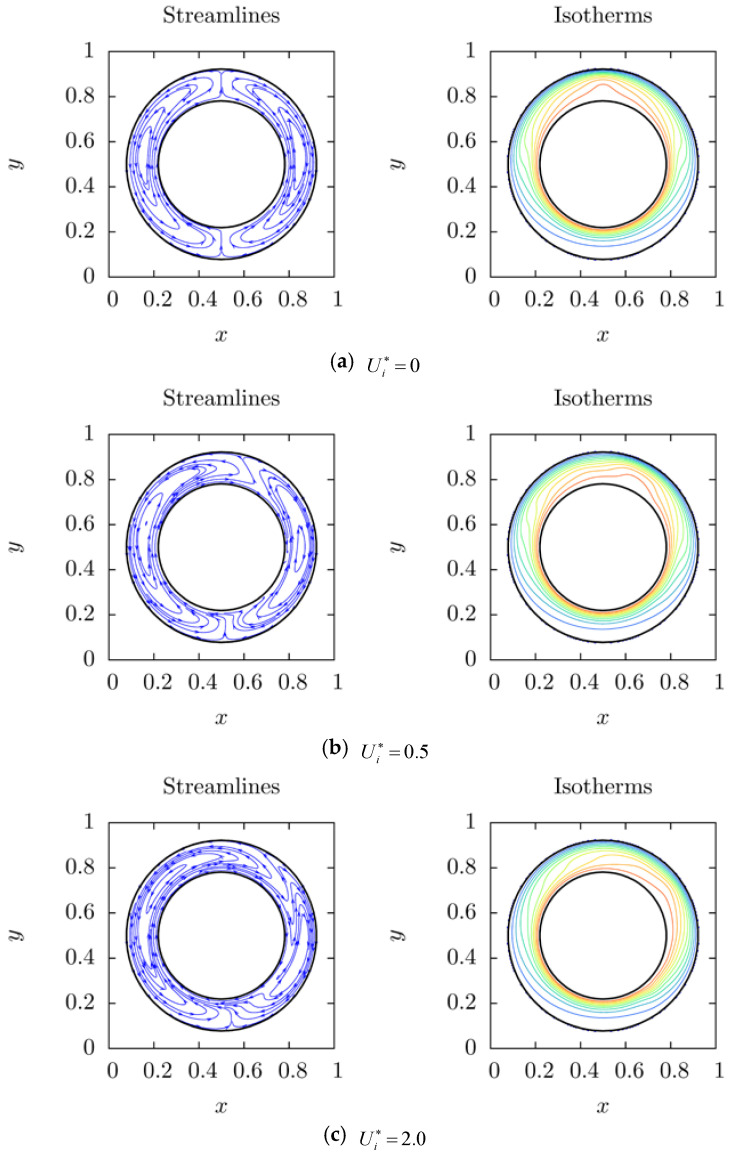

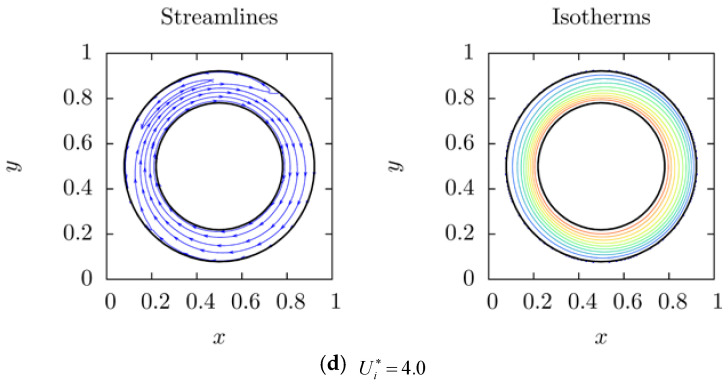
The streamlines and isotherms of rotation effect on 1-peak-state natural convection for $0\leq U_{i}^{*}\leq 4$.

**Figure 7:**
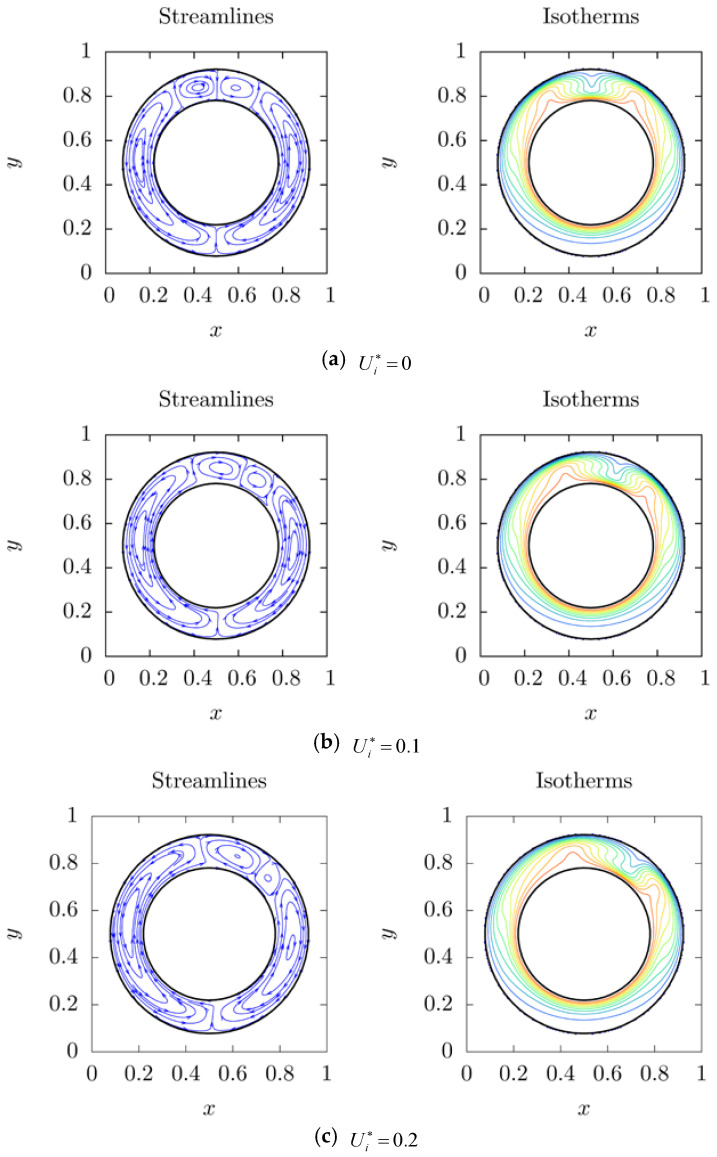

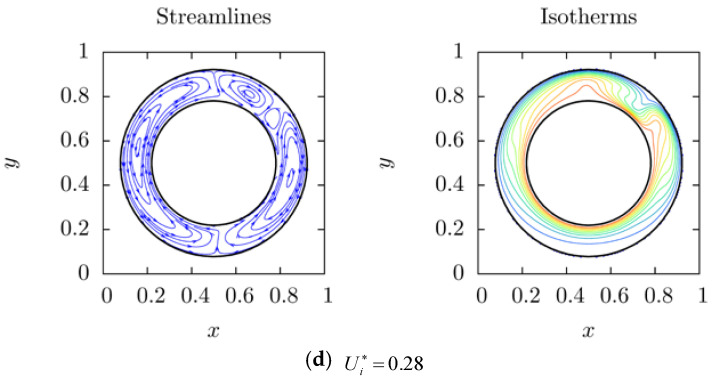
The streamlines and isotherms of rotation effect on 1-peak-state natural convection $0\leq U_{i}^{*}\leq 0.28$.

The effect of linear speed of rotational inner cylinder is further analyzed in this subsection. Since 3-peak-state is an unstable system for rotating inner cylinder, 1-peak-state and 2-peak-state are the only two bifurcation systems discussed in this subsection. The inner cylinder rotates clockwise and its dimensionless linear speed ($U_{i}^{*}$) is ranging from 0 to 4. Figure 6a and Figure 7a show the results obtained from stationary inner cylinder of 1-peak-state and 2-peak-state, respectively. The linear speed is increasingly considered after that.

It is seen that for 1-peak-state, two symmetrical vortices are formed in a stationary annulus as shown in Figure 6a. With increasing linear speed, left vortex is stretched and the right one is compressed, the hot peak moves clockwise and the width of hot peak increases as shown in Figure 6b. One also sees that the separated part of convection at top region deviates from the middle line more obviously than that at bottom region. Left vortex wedges into the right one at top region and compresses the right vortex. The right vortex then approaches the outer cylinder. From Figure 6c, three main convection appear in the annulus. It is obtained that the first convection is the left vortex and the second one is the right vortex. This suggests that the third convection starts from right part of bottom region near the right vortex, clings to the hot inner cylinder, streams around the right vortex and finally back to the right part of bottom region. From Figure 6d, one also sees that the first convection, the left vortex is compressed and is located in the top region, the second convection, and the right main vortex disappears, the third convection is developed into a main convection in the annulus when the dimensionless linear speed is faster than $U_{i}^{*}=4.0$. This mainly implies that the disappearance of the second convection leads to a rapid decrease of the *Nusselt* number after the critical number of dimensionless linear speed, which is between 3 and 4.

For 2-peak-state, a stable range of $0\leq U_{i}^{*}\leq 0.28$ is considered, in which 2-peak-state is stable. The streamlines and isotherms of rotation effect on 2-peak-state are shown in Figure 7, where four dimensionless speed, namely, $U_{i}^{*}$=0, 0.1, 0.2, 0.28 are considered. As shown in Figure 7a, one can see that a pair of main vortices and a pair of small vortices at top region emerge symmetrically in the annulus at stationary condition. From Figure 7b,c, it is seen that as linear speed of the inner cylinder increases, the symmetry of the flow are broken, the left vortices of both pairs are stretched and the right ones are compressed. It is also obtained that two hot peaks move clockwise and the width of left hot peak becomes larger than the right one. Figure 7d shows the result from highest linear speed before critical speed. It is seen that the compressed vortex of the small pair approaches the vertical position of the annulus. Amazingly, it is further found that the compressed small vortex disappears, and the stretched small vortex and the compressed main vortex merge with each other; the system then turns to 1-peak-state as the dimensionless linear speed is larger than a critical number.

**Figure 8:**
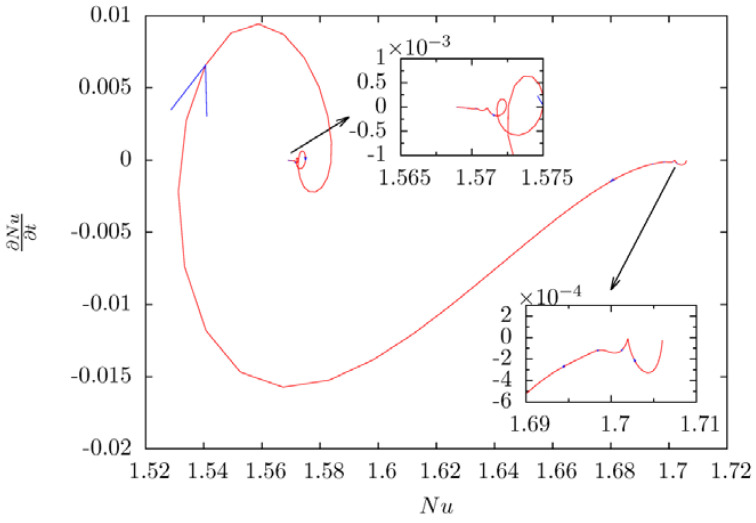
The phase portrait of 2-peak-state turns to 1-peak-state.

Figure 8 illustrates the phase portrait of the progress of 2-peak-state turning to 1-peak-state. In order to show the detail in the phase portrait, final phase trajectory of *Nusselt* number are zoomed and presented in the inset of Figure 8. The steady 2-peak-state flow field with $U_{i}^{*}=0.28$ is used as the initial condition of this simulation, the speed of inner cylinder is increased to 0.29. After the non-equilibrium progress of small vortex disappearing and two vortex merging with each other, as analyzed before. This also suggests that the *Nusselt* number becomes that of steady 1-peak-state with inner cylinder speed equals 0.29 gradually. It is further found that the critical number of dimensionless linear speed is between 0.28 and 0.29, the bifurcation phenomenon disappears and the steady state is 1-peak-state when the linear speed is higher than the critical speed.

**Figure 9:**
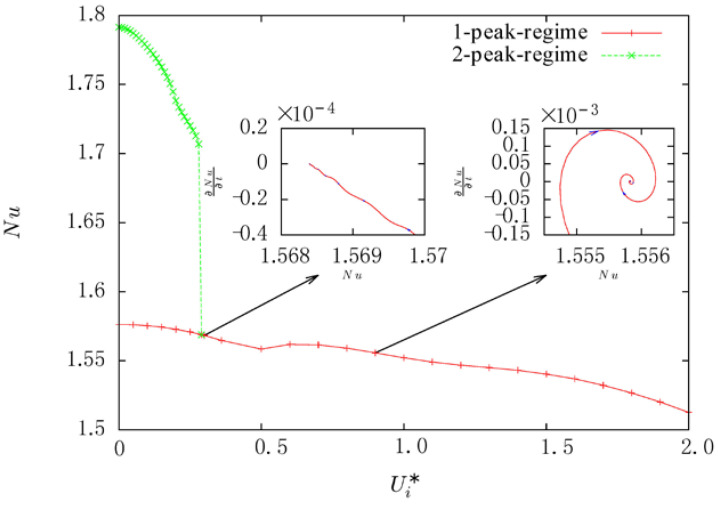
The bifurcation diagram of natural convection in horizontal concentric cylinders with rotating inner cylinder.

As shown in Figure 9, both duel solutions can be obtained till $U_{i}^{*}=0.28$. With the increment of linear speed, the rotation effect leads to the decrement of the average *Nusselt* number, and it is more obvious in 2-peak-state than that in 1-peak-state, which mainly illustrates that 2-peak-state is more sensitive than 1-peak-state. It is obtained that the average *Nusselt* number of 2-peak-state is larger than that of 1-peak-state, i.e., heat convection is more important in 2-peak-state than that in 1-peak-state. It is implied that when $U_{i}^{*}$ is greater than 0.29, 2-peak-state is unstable. A local minimum value of average *Nusselt* number which appears at $U_{i}^{*}=0.5$, indicating two different main mechanisms driving the convection. For $U_{i}^{*}\leq 0.5$, the system is over-damped for $U_{i}^{*}\geq 0.6$, the system is under-damped. Figure 9 shows the phase trajectories of cases at $U_{i}^{*}=0.3$ and $U_{i}^{*}=0.9$ as examples of these two systems. This also suggests that the third convection seems leading to the behavior of under-damped trajectories while the first and the second convection seems leading to the behavior of over-damped trajectories. It is further found that a small increase occurs for the *Nusselt* number, when the third convection is taken control, in which this increment results in the local minimum value of average *Nusselt* number at around $U_{i}^{*}=0.5$.

## 5. Conclusions

In this paper, numerical simulations have been performed to demonstrate the existence of bifurcation phenomena in natural convection in a horizontal annulus to investigate the rotation effect on it by using the thermal IB-LBM. Several conclusions can be summarized as follows.

In the first place, it is validated that numerical results of the present method are well consistent with experimental results. Our results manifest that 1-peak-state is the most stable system, 2-peak-state is more sensitive than 1-peak-state and 3-peak-state is the most sensitive system which will breakdown by a very small perturbation.

After that it is found that the rotation effect on 2-peak-state is more obvious than 1-peak-state, since 2-peak-state is more sensitive than 1-peak-state. It is noted that in the range of the present parameters, 2-peak-state is stable. When the linear speed of inner cylinder exceeds the critical speed, 2-peak-state breakdown and turns to 1-peak-state. From the flow pattern shown by streamlines, one may indicate that the right small vortex is compressed and finally disappears, then the left small vortex and the right main vortex merge with each other and the flow turns to 1-peak-state. Finally, three convections in this system which are affected by different linear speed of inner cylinder were discussed in detail. It is implied that the first and the second convections are contributed by the left and right main vortices, respectively. The third convection is a new vortex under the effect of rotation. With increasing linear speed, left main vortex is stretched and period of the first convection increases. Right main vortex is compressed and the scope of the second convection decreases. The third convection grows with the acceleration of rotation. It is further found that 2-peak-state is stable in the range of 0 ≤ $U_{i}^{*}$ ≤ 0.28, and when the linear speed of inner cylinder exceeds the critical speed, 2-peak-state breakdown and turns to 1-peak-state.

## Figures and Tables

**Table 1:** The grid independence test and comparison for the average Nusselt number. $R_{\beta }=2.6$ and Pr=0.7.

Ra	Grid Size				Experiment [3]
	128×128	256×256	512×512	Extrapolation	
$1\times 10^{2}$	1.034	1.016	1.008	1.002	1.000~1.002
$1\times 10^{3}$	1.101	1.092	1.087	1.081	1.081~1.084
$1\times 10^{4}$	1.955	1.977	1.989	2.003	2.005~2.010
$2\times 10^{4}$	2.346	2.364	2.375	2.392	2.394~2.405
